# Pathophysiological mechanisms of thrombosis in acute and long COVID-19

**DOI:** 10.3389/fimmu.2022.992384

**Published:** 2022-11-16

**Authors:** Haijiao Jing, Xiaoming Wu, Mengqi Xiang, Langjiao Liu, Valerie A. Novakovic, Jialan Shi

**Affiliations:** ^1^ Department of Hematology, The First Hospital, Harbin Medical University, Harbin, China; ^2^ Department of Research, VA Boston Healthcare System, Harvard Medical School, Boston, MA, United States; ^3^ Department of Medical Oncology, Dana-Farber Cancer Institute, Harvard Medical School, Boston, MA, United States

**Keywords:** inflammation, immunothrombosis, anti-inflammatory treatment, antithrombotic therapy, long COVID-19

## Abstract

COVID-19 patients have a high incidence of thrombosis, and thromboembolic complications are associated with severe COVID-19 and high mortality. COVID-19 disease is associated with a hyper-inflammatory response (cytokine storm) mediated by the immune system. However, the role of the inflammatory response in thrombosis remains incompletely understood. In this review, we investigate the crosstalk between inflammation and thrombosis in the context of COVID-19, focusing on the contributions of inflammation to the pathogenesis of thrombosis, and propose combined use of anti-inflammatory and anticoagulant therapeutics. Under inflammatory conditions, the interactions between neutrophils and platelets, platelet activation, monocyte tissue factor expression, microparticle release, and phosphatidylserine (PS) externalization as well as complement activation are collectively involved in immune-thrombosis. Inflammation results in the activation and apoptosis of blood cells, leading to microparticle release and PS externalization on blood cells and microparticles, which significantly enhances the catalytic efficiency of the tenase and prothrombinase complexes, and promotes thrombin-mediated fibrin generation and local blood clot formation. Given the risk of thrombosis in the COVID-19, the importance of antithrombotic therapies has been generally recognized, but certain deficiencies and treatment gaps in remain. Antiplatelet drugs are not in combination with anticoagulant treatments, thus fail to dampen platelet procoagulant activity. Current treatments also do not propose an optimal time for anticoagulation. The efficacy of anticoagulant treatments depends on the time of therapy initiation. The best time for antithrombotic therapy is as early as possible after diagnosis, ideally in the early stage of the disease. We also elaborate on the possible mechanisms of long COVID thromboembolic complications, including persistent inflammation, endothelial injury and dysfunction, and coagulation abnormalities. The above-mentioned contents provide therapeutic strategies for COVID-19 patients and further improve patient outcomes.

## Introduction

Recent studies demonstrate that the incidences of venous thromboembolism (VTE) and pulmonary embolism in hospitalized patients with COVID-19 are 17% and 7.1%, respectively, while the incidence of major bleeding is only 3.9% (1, 2). Systematic reviews have shown a higher incidence of VTE at 28% in COVID-19 patients admitted to the ICU (3, 4). Autopsy studies on COVID-19 patients have reported thrombi composed of platelets and fibrin in pulmonary vascular (5, 6). COVID-19 patients exhibit unique coagulopathy, characterized by thrombus formation with elevated levels of cytokines, including tumor necrosis factor (TNF)-α, interleukin (IL)-8, and IL-6, among others (7). Inflammatory molecules and immune cells are considered the main drivers for thrombosis in COVID-19 patients. Transient increases of thrombotic risk induced by viruses (including SARS-CoV-2) indicates that subsequent host immune response is a major trigger of vascular thromboembolic events, rather than the infection itself, independent of the pathogen species (8). Even with effective antithrombotic drugs, thrombotic events cannot be completely prevented, which suggests the presence of other unresolved mechanisms that result in under-treatment, such as inflammation. The above evidence supports the hypothesis that COVID-19-associated thrombosis is immunothrombosis triggered by inflammation. Immunothrombosis is described as the immune system triggering coagulation to form thrombosis that prevents pathogen transmission, with immune cells (neutrophils, etc.), NETs, and platelets playing important roles in this process (9, 10). Molecular interactions between inflammation and coagulation have been gradually confirmed. Inflammation and the coagulation cascade form complex networks that work to counterbalance the body’s response to pathogen invasion and tissue damage, and maintain homeostasis (11). Inflammation is a defense mechanism triggered by the immune system in response to injury or infection and is characterized by increased local blood flow, leukocyte recruitment, and the release of cytokines and chemokines, which can promote pathogen clearance. Inflammation also induces increased expression of adhesion molecules on endothelium, the subsequent interactions between leukocytes and endothelial cells (ECs) can lead to immune cell activation, thereby forming immunothrombosis at the injured area (12–14). Immunothrombosis can further limit systemic transmission of pathogens *via* the bloodstream and is an emergent mechanism for the host to inhibit infection (15). However, in COVID-19, thrombosis is related to disease progression and is associated with increased mortality (16–18). Therefore, to improve prognosis, early prevention and treatment of thrombosis is critical for COVID-19 patients.

## Mechanisms of inflammation in COVID-19

Severe COVID-19 is the result of intense interactions between SARS-CoV-2 and the host, causing progressive damage to tissues and organs (19). Spike (S) protein in the viral envelope exists as homotrimers, including the receptor-binding S1 subunit and the membrane fusion S2 subunit. The receptor-binding domain (RBD) of the virus S1 subunit binds to ACE2 on the epithelium of the nasopharynx. S1/S2 or S2 site hydrolysis by transmembrane protease serine 2 (TMPRSS2) is required for the activation of RBD, causing a confirmation change in the S protein which catalyzes virus-host membrane fusion (20, 21). This is followed by viral RNA replication, particle assembly and release (22). SARS-CoV-2 viral RNA is recognized by the innate immune cells *via* pattern recognition receptors (PRRs), such as Toll-like receptors (TLR, including TLR3, 7, 8), as well as the NOD-like receptors (NLR). Then, the activated down-stream transcription factor, NF-κB, induces the generation of pro-inflammatory cytokines. These cytokines contribute to the formation of the cytokine storm. Additionally, SARS-CoV-2 has been reported to exacerbate the inflammatory response of myeloid cells through C-type lectin receptors and Tweety family members (23). This non-TLR pathway contributes to virus-induced cytokine storm. C-type lectins not only contribute to cytokine storm. Among the members of the human C-type lectin family, dendritic cell-specific intercellular adhesion molecule-3-grabbing non-integrin (DC-SIGN), L-SIGN, LSECtin and Syk-coupled C-type lectin member 5A (CLEC5A) and CLEC2 are critical in virus-induced NET formation (24). Dengue virus (DV) activates platelets to release extracellular vesicles (EVs) by CLEC2, including exosomes (EXOs) and microvesicles or microparticles (MVs or MPs). DV induced EXOs (DV-EXOs) and MVs (DV-MVs) further activate neutrophils and macrophages by CLEC5A and TLR2, thereby inducing NET formation and pro-inflammatory cytokine release. Consequently, the blockade of C-type lectins is a promising strategy to attenuate virus-induced NETs formation (25). In addition to DV, influenza A virus (IAV, H5N1) can also activate platelets to release EVs, thereby enhancing NET formation. A recent study shows that SARS-CoV-2 viral proteins induce NET formation *via* a Syk-dependent pathway of downstream signaling molecules of C-type lectin receptors (26). Further study shows that EVs released from SARS-CoV-2-activated platelets induce robust formation of NET *via* CLEC5A and TLR2, which in turn enhance thrombosis (27).

Interferon regulatory factor 3/7 (IRF3/7) is activated to enhance the transcription of interferon, which contributes to the host antiviral response. Growing evidence indicates protective type I interferon responses of COVID-19 patients are significantly reduced, which further leads to disease progression (19). Siglecs (sialic acid binding immunoglobulin-like lectins) are receptors of immune cell membranes and can interact with sialoglycans on viruses. Typically, most siglecs with structural domains containing the immunoreceptor tyrosine-based inhibitory motif (ITIM) mainly exert a negative regulatory role to prevent excessive immune responses. Sialoglycans on the SARS-CoV-2 envelope interact with siglecs on host cells to downregulate host immunity to clear virus, thereby aggravating viral infection (28). In the lung parenchyma, the virus damages the alveolar epithelial cells, releasing damage-associated molecular patterns (DAMPs), including nucleosomes, histone, and human high mobility group protein 1 (HMGB1), which activate innate immune cells to release chemokines and cytokines, such as TNF-α, IL-6, IL-1β, etc. Then, these cytokines and chemokines recruit more innate immune cells (neutrophils, monocytes, macrophages, natural killer cells) and activate adaptive immune cells (CD4^+^, CD8^+^ T) to also produce cytokines, ultimately leading to a cytokine storm (28, 29). The build-up of cytokines aggravates the damage, necrosis, and even sloughing of the alveolar epithelium, and forms a positive feedback loop of epithelial damage. Myeloid cells are the main source of inflammatory dysregulation in the lung tissue of COVID-19 patients (30).

## Inflammatory markers in COVID-19 patients

Increased levels of IL-8, IL-6, monocyte chemoattractant protein-1, and TNF-α are observed in the plasma of COVID-19 patients. Elevated TNF-α, in particular, is a marker of poor prognosis and may lead to endothelial dysfunction in patients with COVID-19 (31, 32). IL-6 plays an essential role in driving pathological inflammatory responses, and is also associated with more severe outcomes. In a study of 2782 COVID-19 patients, high levels of C reactive protein (CRP) correlate with progression to severe cases, acute kidney injury, and all-cause mortality (33, 34). CRP levels display a positive relationship with COVID-19 severity and have been demonstrated to predict the risk of thrombosis (33, 35).

## The normal physiological function of endothelium

Virchow’s triad describes the contributing factors to thrombus formation: damaged vessel walls, alterations in blood flow, and changed blood composition (hypercoagulability), and is fundamental to understanding the pathophysiology of venous thrombosis (36, 37). Damage to and subsequent activation of ECs are the first and critical steps for thrombosis. Intact ECs maintain blood fluidity and vascular homeostasis primarily through anti-inflammatory and anticoagulant effects.

Endothelial barrier function is dependent on stable cell-cell junctions, including adherens and tight junctions (38). ACE2 on the endothelium generates angiotensin (1–7), which binds to Mas1 receptor, modulating anti-inflammatory and antithrombotic signaling pathways (39, 40). Heparan sulfate within the glycocalyx binds to and activates antithrombin, and thus limits thrombosis on the endothelium. Additionally, ECs also support natural anticoagulant and antifibrinolytic substances, including thrombomodulin (TM), endothelial protein C receptor (EPCR), and tissue factor pathway inhibitor (TFPI) (41, 42). TM on the endothelial surface binds to thrombin, and then forms a complex with ERCP, and converts protein C to its active form (activated protein C, APC). APC and its cofactor protein S inactivate FVa and FVIIIa, thereby reducing thrombin generation (43, 44). TFPI, an endogenous serine protease inhibitor, can directly inhibit FXa and FVIIa/TF complex, and thereby exert anticoagulant effects *via* the extrinsic pathway of coagulation. ECs can secrete tissue plasminogen activator (t-PA) and urokinase-type plasminogen activator (u-PA), express the u-PA receptor (uPAR), and provide potent fibrinolytic ability. The inhibitors of complement cascade can protect quiescent ECs from complement deposition. Quiescent endothelium can also prevent platelet adhesion by the expression of CD39 and the release of prostaglandin I2 (PGI2) and nitric oxide (NO), thereby dampening platelet activation (45, 46). NO and PGI2 also exert other cytoprotective effects. NO can suppress the recruitment of leukocytes to the vessel wall through reducing the expression of chemokines and the transcription of adhesion molecules on the endothelial surface. PGI2 can reduce leucocyte adhesion, activation, and extravasation to attenuate inflammatory responses (47).

## Inflammation-driven immunothrombosis

Inflammatory stimuli can overwhelm the delicate antithrombotic balance described above, promote inflammatory environments, and eventually lead to thrombus formation (48, 49). Cytokine signaling, hypoxia, tissue damage, and DAMPs trigger the transition of ECs from an anticoagulant to a pro-coagulant phenotype (11). Under inflammatory conditions, the loss of vascular integrity, reduced expression of antithrombotic molecules, the interactions between neutrophils and platelets, platelet activation, TF expression on monocytes, microparticle release, and inhibition of fibrinolysis lead to immunothrombosis (50, 51).

## The interaction between platelet and neutrophil

DAMPs and cytokines induce an increased release of vWF (von Willebrand factor) from Weibel-Palade bodies to ECs surface. Platelets bind to vWF on the ECs through glycoprotein Ib (GP Ib). ADAMTS13 (a disintegrin and metalloprotease with thrombospondin type-1 repeats, member-13) regulates the size of vWF multimers and inhibits platelet-rich thrombosis by cleavage of vWF (52, 53). Plasma ADAMTS13 levels are decreased in patients with severe COVID-19 compared to those with mild disease, and reduced ADAMTS13 levels are associated with increased mortality rates (52). The elevated vWF and decreased ADAMTS13 levels are the potent predictors of adverse outcomes in severe COVID-19 patients (51, 54). ECs perturbation, increased release of vWF, and relatively insufficient vWF cleavage owing to the deficiency of ADAMTS13 are responsible for increased interactions between platelets and the vessel wall to cause thrombotic microangiopathies.

Platelets in COVID-19 patients are characterized by hyperreactivity (increased aggregation, increased expression of CD40 and p-selectin). In posthoc analyses of 300 patients with COVID-19, plasma p-selectin levels at admission were strongly associated with the subsequent diagnosis of venous thromboembolism (55). Mean platelet volume (a biomarker of platelet hyperactivity) was related to the occurrence of thrombosis and disease severity (56). In a meta-analysis of 15 studies, the presence of larger, more immature platelets was associated with an increased risk of thrombosis and all-cause mortality (57). Additionally, Analyses of tissue and blood from COVID-19 patients revealed that SARS-CoV-2 viral virions entered megakaryocytes and platelets, which was related to alterations in the platelet transcriptome and activation profile (56).

Cytokines result in increased expression of adhesion molecules on the surface of ECs including P-selectin and E-selectin, which favor rapid recruitment of leukocytes to the site of injury and inflammation (11). Although leukocytes are not typical parts of the coagulation system, they are essential for immunothrombosis, and contribute to the initiation of coagulation by ECs. Neutrophils bind to chemokine (C-X-C motif) ligand 1 and P-selectin on ECs by (C-X-C motif) chemokine receptor 2 and P-selectin glycoprotein ligand-1, respectively, whereas platelets attach to EC-bound vWF. Bound platelets are then activated by SARS-CoV-2 and release HMGB1-bearing extracellular vesicles, inducing neutrophils to generate neutrophil extracellular traps (NETs), web-like structures of which entrap blood cells and coagulation factors (58). In turn, active substances such as antimicrobial peptides released by NETs can further activate platelets. In this way, NETs provide scaffolds for thrombosis. This creates a positive feedback loop, and the crosslinking of platelets with NETs components results in immunothrombosis. The interactions of activated platelets with immune cells stimulate the coagulation system, forming an intertwined process bridging thrombotic and inflammatory pathways (59). Indeed, studies have shown that the levels of neutrophil–platelet aggregates in the blood of COVID-19 patients are elevated (60). Additionally, among COVID-19 patients requiring hospitalization, those admitted to the ICU have the highest levels of platelet-leukocyte aggregation, suggesting that platelet-leukocyte aggregation may be a surrogate marker of COVID-19 severity (60, 61).

NETs are an important interface between inflammation and thrombosis. NETs provide scaffolds for the activated coagulation system and link the immune and coagulation systems by immunothrombosis. NETs can bind to TF, thereby promoting the generation of fibrin. The cell-free DNA released in NETs provides a negatively charged surface to allow FXII binding and activation of the contact (intrinsic) coagulation pathway, resulting in microvascular occlusion (62, 63). Additionally, neutrophil elastase (NE) and myeloperoxidase, two other components of NETs, can cleave and inactivate natural anticoagulants (TFPI and TM), thereby leading to a procoagulant shift of the hemostatic balance and local clot formation (54, 64).

**Figure 1:**
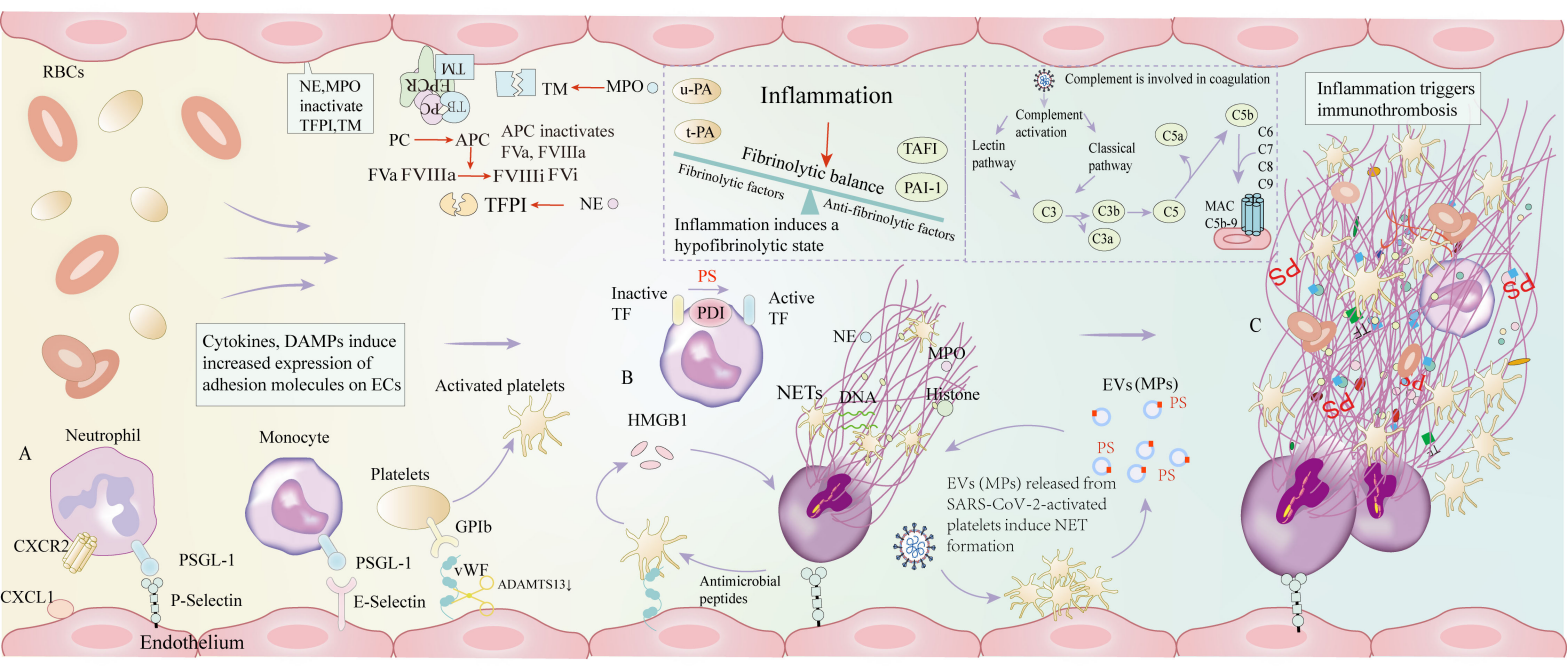
The mechanisms of immunothrombosis in COVID-19 patients. **(A)** Inflammation induces immune cell recruitment and increased expression of adhesion molecules, including P-selectin, E-selectin, and vWF. **(B)** Activated platelets activate neutrophils and generate NETs through the release of HMGB1, and the components of NETs, antimicrobial peptides, further induce platelet activation, forming a positive feedback activation. NE and MPO can inactivate TFPI and TM, thereby inhibiting anticoagulant function. Monocytes exert pro-coagulant effects by converting inactive TF to active TF, which is required for PS and PDI. EVs (MPs) are mainly derived from SARS-CoV-2-activated platelets. Platelet-derived EVs (MPs) are responsible for robust NET formation, which plays a critical role in COVID-19 thrombosis. Activated platelets and platelet-derived EVs are markers and central mediators of COVID-19 immunothrombosis. Under inflammatory settings, elevated levels of thrombin-activatable fibrinolysis inhibitor and PAI-1 surpass the role of u-PA and t-PA, thereby resulting in inhibition of fibrinolysis. SARS-CoV-2 induces complement activation, which is involved in coagulation. **(C)** The above processes eventually cause immunothrombosis. vWF: von Willebrand factor; NETs: Neutrophil extracellular traps; HMGB1: human high mobility group protein 1; NE: Neutrophil elastase; MPO: Myeloperoxidase; TFPI: tissue factor pathway inhibitor; TM: thrombomodulin; TF: tissue factor; PS: Phosphatidylserine; PDI: Protein disulfide isomerase; EVs: Extracellular vesicles; MPs: Microparticles; PAI-1: plasminogen activator inhibitor; u-PA: urokinase-type plasminogen activator; t-PA: tissue plasminogen activator; SARS-CoV-2: severe acute respiratory syndrome coronavirus 2; CXCL-1: chemokine (C-X-C motif) ligand 1; CXCR2: C-X-C motif chemokine receptor 2; PSGL-1: P-selectin glycoprotein ligand-1.

The generation of NETs contributes to adverse coagulation function and immunothrombosis. The components of NETs were observed in serum, plasma, and post-mortem specimens of COVID-19 patients (15, 65). The levels of DNA, myeloperoxidase (MPO)-DNA complexes and citrullinated histones (H3Cit) increased two-fold in COVID-19 patients compared to healthy controls (65). Plasma levels of MPO-DNA complexes and H3Cit correlated with COVID-19 not only with thrombotic events but also with disease severity (66, 67). A prospective cohort study of the autopsy showed that 10 of 21 COVID-19 patients presented with neutrophil embolism (68). Histopathology of the lung and other organs consistently indicated that aggregated NETs led to microvascular obstruction associated with the disruption of the endothelium (33). These observations highlight the potential relationship between NETs and persistent COVID-19 thrombosis (**Figure 1**).

## Monocytes TF expression

Inflammation can induce the recruitment of monocytes, which bind to ECs through E-selectin. Monocytes initiate coagulation by the expression of TF and enhance thromboinflammation by the activation of the inflammasome. TF expression is triggered by pyroptosis, which is a cell death process dependent on caspase-1, involving the formation of pores in the cell membrane and the release of inflammation mediators (IL-1β and IL-18). TF is the primary activator of the extrinsic coagulation pathway that functions by forming a complex with circulating FVIIa and enhancing the activation of FIX and FX, which goes on to initiate coagulation (10, 69). Thus, TF exposure induces increased thrombin generation, which leads to the formation of fibrin clots. Protein disulfide isomerase (PDI) is an important regulator of extracellular disulfide-exchange and is associated with TF coagulant activity on the cell surface (70). PDI released at the site of vascular lesions contributes to TF activation by converting TF from a nonfunctional encrypted state to its active form (71, 72). The release of PDI is tightly regulated to prevent continuous thrombosis under physiological conditions. Adherent platelets and impaired vascular wall can promote thromboinflammation by releasing PDI. Besides PDI, phosphatidylserine (PS) exposure (externalization) can also directly promote TF-dependent fibrin formation, as explained below (**Figure 1**).

## EVS release and PS exposure

Activated platelets and platelet-derived extracellular vesicles (EVs) are markers and central mediators of COVID-19 immunothrombosis. The levels of platelet-derived EVs were higher in COVID-19 patients requiring hospitalization than in those not requiring hospitalization (73). Platelet-derived as well as endothelium-derived EVs are responsible for NET formation and endothelial cell death (10, 74). Robust formation of NET induced by platelet-derived EVs further enhances immunothrombosis (59). Additionally, increased levels of TF^+^ EVs in the plasma of COVID-19 patients are related to thrombin generation, the severity of respiratory condition and mortality risk (75, 76).

**Figure 2:**
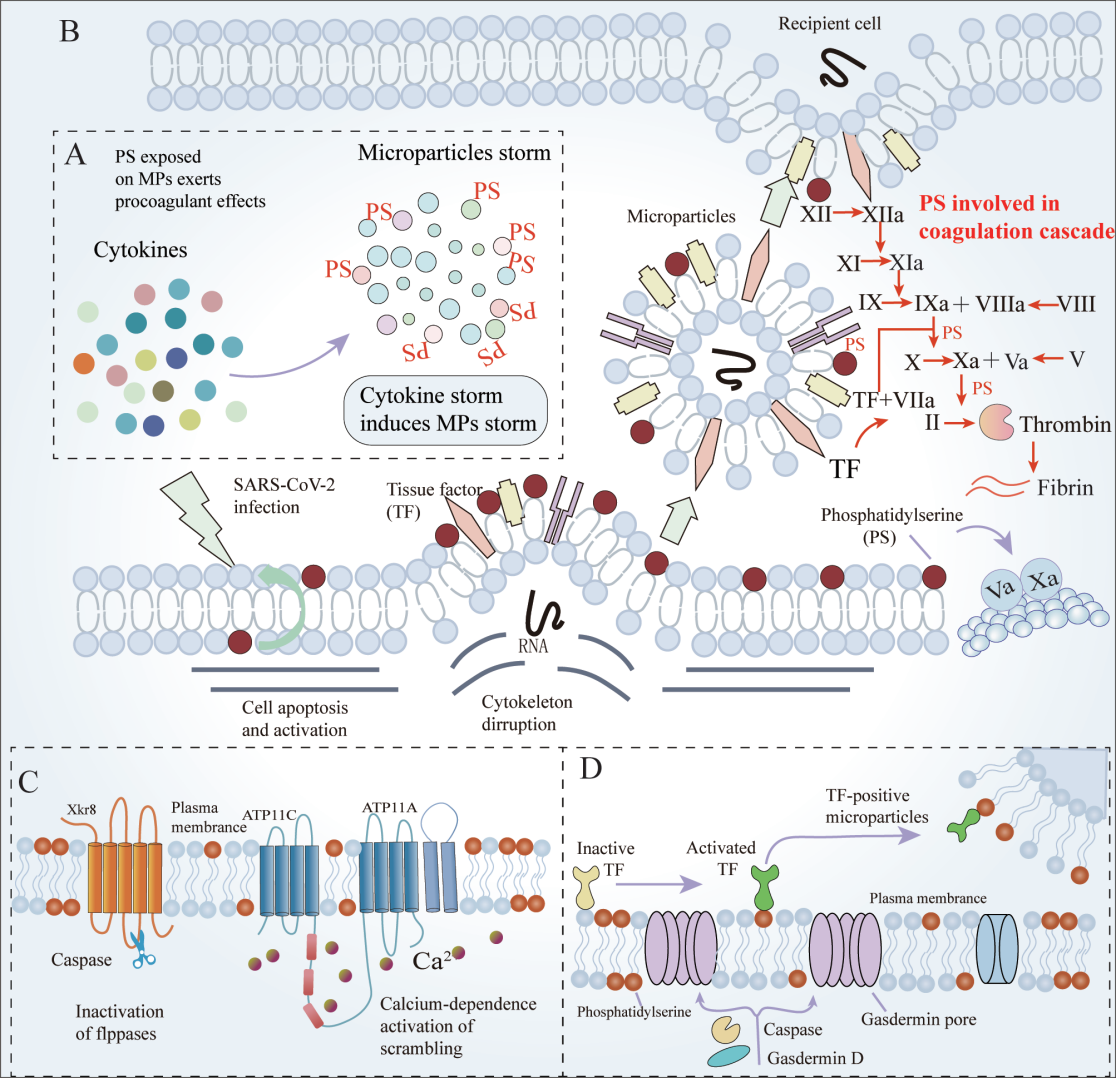
The mechanisms of MPs and PS involved in coagulation. **(A)** High levels of cytokines induce the apoptosis of blood cells, and ECs, release MPs (mainly derived from platelets), and exert pro-coagulant effects. Additionally, platelet activation is the primary source of pro-coagulant MPs. **(B)** PS is exposed on MPs in case of cell apoptosis and activation, and then is involved in coagulation by the coagulation cascade **(C)** Caspase-dependent Xkr8 activation and calcium-dependent P4-ATPase inactivation are responsible for PS externalization. **(D)** Coagulation activity of TF and the release of MPs bearing TF require caspase-dependent gasdermin D-mediated PS externalization. ECs, Endothelial cells; MPs, microparticles; PS, phosphatidylserine; Xkr8, Xk-related protein 8; TF, Tissue factor.

MPs (a kind of EVs) are enveloped by a phospholipid bilayer membrane and released from apoptotic or activated cells. Cytokines within the microvasculature induce the apoptosis of blood cells and ECs, causing the release of MPs, which exert pro-coagulant effects. Additionally, platelets activated by SARS-CoV-2 are the primary source of pro-coagulant MPs. Several studies have mentioned that the MPs released from virus-activated platelets cause robust NET formation and inflammatory reactions (25, 74). Strong evidence suggests that MPs are procoagulant due to the exposure of PS. Type IV P-type ATPases (P4 ATPases) are flippases that transport specific lipids (PS) from the inner to the outer leaflet of the membrane bilayer. Transmembrane protein 16F (TMEM16F) and Xk-related protein 8 (Xkr8) are two scramblases involved in the process of PS transport (77, 78). PS on MPs moves to the outside layers through caspase-dependent Xkr8 activation and calcium-dependent P4-ATPase inactivation, which is referred to as PS externalization. PS provides binding sites for coagulation factors V and VIII. TMEM16F-mediated PS externalization on the surface of monocytes triggers the caspase-11 and gasdermin D pathways, which regulates the coagulation activity of TF and promotes the release of MPs bearing TF (79) (**Figure 2**). MPs release and PS externalization are the main drivers of COVID-19-associated pulmonary coagulopathy. First, as the severity of the disease worsens, more and a larger diversity of apoptotic cells release MPs, including red blood cells, platelets, monocytes/macrophages, and ECs. PS on MPs is a potent modulator of the human blood-clotting system. Secondly, PS initiates the extrinsic coagulation pathway by decrypting TF. PS enhances the catalytic efficiency of the tenase and prothrombinase complexes by providing scaffolds for these coagulation factors (80, 81) (**Figure 2**). Elevated platelet-derived MPs in circulation correlate with an increased risk of thromboembolic complications, leading to increased severity and mortality of COVID-19 (82, 83). The number of PS+ peripheral blood mononuclear cells (PBMCs) in blood samples from COVID-19 patients is abnormally high, and becomes a new parameter that correlates strongly with disease severity. Compared to those without thrombosis, COVID-19 ICU patients with thromboembolic events had significantly increased platelet PS externalization, an apoptotic marker, suggesting the link between venous thromboembolism and PS externalization (84).

## Complement activation

SARS-CoV-2 activates the complement cascade by the classical and the lectin pathways (85). The complement system is a major contributor to inflammation and thrombosis. C3a and C5a recruit and activate neutrophils and monocytes to release pro-inflammatory cytokines (IL-6, IL-8). C5a can upregulate the expression of TF on neutrophils and ECs, which in turn exerts a procoagulant effect (86). C5a can also inhibit fibrinolysis by increased release of plasminogen activator inhibitor 1 (PAI-1) from mast cells and basophils (86, 87). In an *in vitro* system, the plasma of COVID-19 patients induces complement activation to trigger NETosis in a C5a-dependent manner (88). Membrane attack complex (MAC) induces ECs to actively secrete vWF and assemble the prothrombinase complex (89). Multiple complement-centric interconnections between inflammatory pathways and prothrombotic mechanisms act synergistically to suppress pathogens. Complement factors (C1q and C3), and MAC can activate platelets. In turn, platelets provide a surface for complement activation, and enhance inflammatory function of neutrophils by binding to complement (90, 91). Furthermore, platelets capture pathogens in a C3- and GP Ibα-dependent manner, transport them to antigen-presenting dendritic cells, and thereby initiate adaptive immune responses (92). Complement factors (C5b-9 and C4b) and MASP2 are shown in pulmonary microvascular of severe COVID-19 patients (15, 93). According to autopsy outcomes, complement is related to microvascular occlusion in the context of severe COVID-19 (**Figure 1**).

## Fibrinolytic inhibition

When thrombus occurs in the vascular intima, ECs can express plasminogen activators including t-PA and u-PA, thereby contributing to the generation of endogenous fibrinolytic substances. Activated ECs can also produce PAI-1, which antagonizes u-PA and t-PA, thereby maintaining fibrinolytic balance. But inflammation can result in a hypo-fibrinolytic state in COVID-19 patients. Studies show that low levels of plasminogen are significantly related to inflammatory markers (CRP, procalcitonin, and IL-6) (94, 95). Inflammation itself can promote the local release of t-PA and PAI-1 from ECs, suggesting that increased PAI-1 levels may affect fibrinolysis more than elevated t-PA levels which are simultaneously presented in COVID-19 patients (96, 97). In addition, elevated levels of thrombin-activatable fibrinolysis inhibitor (TAFI) and PAI-1 surpass the role of u-PA and t-PA, thereby resulting in inhibition of fibrinolytic function (98) (**Figure 1**).

## The crosstalk between inflammation and coagulation

The crosstalk between inflammation and coagulation mainly depends on three coagulation factors: TF, thrombin, and FXII. In addition to promoting coagulation, TF also initiates inflammatory pathways by G protein-coupled protease-activated receptors (PARs), which are differentially expressed on ECs, platelets, and leukocytes (99). The activation of PARs initiates multiple inflammatory signals including cytokines and growth factors, which induce the expression of adhesion molecules. TF is the pivotal trigger of coagulation activation, and the initial events leading to its exposure in circulating blood propel an escalating feedback loop between inflammation and coagulation. If thromboinflammation loses its natural brakes, it may result in overwhelming thrombotic and inflammatory injury. The partial pro-inflammatory effects of thrombin are mediated by ECs stimulation. Thrombin activates ECs primarily by PAR1 proteolysis, and induces increased P-selectin expression and mobilization of Weibel-Palade bodies (41, 100). Thrombin cleaves PARs on platelets, triggers the release of granular contents, such as ADP, CD40 ligand, and multiple pro-inflammatory mediators (101, 102). Thrombin also stimulates platelets, which appear to enhance rapid and efficient recruitment of neutrophils to localized endothelial injury sites (103). The activation of the contact system of coagulation can also release pro-inflammatory mediators including bradykinin (BK) (104). Proteins of the contact system (FXII, high molecular weight kininogen (HK) and plasma kallikrein (PK)) can assemble on the cell surface to form a complex, which binds to soluble contact activator (including poly P) carried by the cell surface, resulting in FXII activation. FXIIa, in turn, triggers thrombus formation by the intrinsic coagulation pathway. FXIIa can also activate PK, and generate BK from HK. BK and its active metabolite Des-Arg 9-BK exert pro-inflammatory effects.

## Long COVID

Long COVID or post-acute COVID-19 syndrome (PACS) is an emerging syndrome worldwide, characterized by the persistence of signs and symptoms of COVID-19 for more than four weeks after infection (105). The symptoms of long COVID mainly include fatigue, dyspnea, reduced exercise tolerance, chest pain, and thrombotic complications (106, 107). About 10% of COVID-19 patients develop persistent or relapsing and remitting symptoms beyond 4 to 12 weeks following infection (106). Studies show that 4.5%-36.6% of COVID-19 patients remain symptomatic greater than 3 months after infection (108–110). This percentage is as high as 76% among patients requiring hospitalization during the infection (111, 112). A single-center report of 163 patients who did not undergo post-discharge thromboprophylaxis found that the cumulative incidence of thrombosis 30 days after discharge was 2.5%, including pulmonary embolism and ischemic stroke, etc (113). Prospective studies of 6 weeks post-discharge follow up assessing d-dimer levels and venous ultrasound showed 8% of COVID-19 patients receiving thromboprophylaxis after hospital discharge (114, 115). Currently, vaccination is the most effective preventative measure for severe COVID-19 symptoms. Since the rollout of COVID-19 vaccines, a global vaccination campaign has been carried out. Studies have shown that receiving at least one dose of COVID-19 vaccine significantly reduces the risk of ICU stay, and hypoxemia, but not some outcomes of long COVID. Receiving two doses of vaccine (i.e. BNT162b2 Pfizer/BioNTech, mRNA-1273 Moderna, or Ad26.COV2. S Janssen) is associated with a lower risk of most outcomes (116, 117). Some studies also suggest that vaccine administered pre-infection can only provide partial protection during the acute stage of the disease, but not reduce the long-term consequences of SARS-CoV-2 infection (which may include thrombosis) (118, 119). Therefore, we will focus on the thromboembolic complications of Long COVID, which can lead to serious consequences for COVID-19 patients.

Despite the increasing awareness of Long-COVID, the potential pathophysiological mechanisms of it remain poorly characterized, and may correlate with several factors (120, 121). Proposed causes include lingering viruses, chronic inflammation, or microscopic thrombi (122, 123). Long COVID may be caused by a combination of multiple factors (124–126). In the following section, we summarize the possible mechanisms of long COVID thrombosis, including endothelial lesions, coagulation abnormalities and persistent inflammation.

In the acute phase of COVID-19, apart from mediating inflammatory immune responses, SARS-CoV-2 can directly damage ECs by binding to AEC2 (127). The damage of ECs leads to endothelial activation, loss of barrier function, and increased permeability followed by microvascular leakage (128–130). Endothelial damage can induce diffuse and systemic endothelial dysfunction, and activate multiple immune-mediated inflammatory pathways and thrombus formation, thereby resulting in severe multi-organ involvement and subsequent increased mortality (131, 132). SARS-CoV-2 has also been detected in ECs, and elevated levels of circulating endothelial cells (CECs, a biomarker of vascular injury) can be found in patients admitted for COVID-19 (124, 125, 133). However, some pathological processes persist even when SARS-CoV-2 is no longer detectable. Elevated markers of endothelial lesions and coagulation are observed in a significant proportion of convalescent patients, suggesting infection may induce chronic coagulopathy, endotheliitis, and microvascular lesions, accompanied by microthrombi formation (126, 134).

## Endothelial damage and dysfunction

In studies of COVID-19 patients enrolled in medical wards or intensive care unit (ICU), the marked endothelial dysfunction gradually improved at the 6 month follow-up, but remained impaired compared to healthy controls (135, 136). The levels of CECs in patients recovered from COVID-19 were elevated, which may be driven by activated T lymphocyte-related cytokines. Furthermore, increased expression of ICAM1, SELP, and CX3CL1 on CECs may indicate that ECs from the site of vascular injury were in pro-inflammatory and procoagulant states (137). In a study of 50 patients with SARS-CoV-2 infection, the highest level of soluble thrombomodulin (14.4 ng/ml) was observed in a patient who did not require hospitalization, indicating persistent endothelial lesions in convalescence were not exclusive to those who exhibited severe COVID-19 (138). During the post-infection phase, flow-mediated peripheral arterial dilation was significantly decreased in COVID-19 patients, suggesting the presence of endothelial dysfunction (139). Cross-sectional studies assessing vascular function 3-4 weeks following SARS-CoV-2 infection showed a significant decrease in systemic vascular function and increased arterial stiffness in SARS-CoV-2-positive participants (135, 140). Overall, results from these studies show persistent endothelial lesions are common in the convalescent phase of COVID-19. The normal endothelium plays an important role in preventing thrombosis by expressing antiplatelet and anticoagulant substances to block platelet aggregation and fibrin clot formation as well as inhibiting the adhesion of coagulation proteins (128, 141). It has been previously demonstrated that endothelial dysfunction is associated with an increased risk of thrombosis (142, 143). Consequently, long COVID thrombosis may be secondary to endothelial damage and subsequent endothelial dysfunction.

## Endothelial activation and coagulation abnormalities

Recent studies have suggested that persistent endothelial activation is commonly observed in convalescent COVID-19 patients (138, 139). Especially, several groups have reported consistently increased levels of endogenous thrombin potential (ETP) and D-dimer in COVID-19 patients 4-12 months following acute infection (138, 144, 145). Significantly elevated peak thrombin, and a shorter time to reach the peak of thrombin formation are also seen in convalescent COVID-19 patients (138, 146). Interestingly, the percentage of intermediate monocytes is associated with elevated ETP and peak thrombin (146). In a cohort of convalescent patients, plasma FVIII: C levels were still significantly higher than in controls. In the same study, reduced ADAMTS13 as well as elevated vWF: Ag levels mean that the vWF: Ag/ADAMTS13 ratio dramatically increased in patients recovered from COVID-19 (107). Studies indicated that markers of platelet activation returned to normal at 2-3 months post-discharge (147, 148). Notably, circulating NETs biomarkers reverted towards normal levels around 4 months following infection compared to healthy controls (149, 150). A series of complex events in the vasculature including endothelial activation, NET formation, vWF secretion, and blood cell adhesion and aggregation collectively mediate immunothrombosis. Therefore, persistent EC activation and increased pro-coagulant substances in convalescence may be linked to long COVID thromboembolic complications.

## Persistent inflammation

Researchers have found the evidence of SARS-CoV-2 in peripheral organs of the body, especially the intestine, for several months following viral clearance from the respiratory airways (151). Weeks to months after the disappearance of acute symptoms, viral RNA and protein antigens can be detected in the central nervous system, intestines, and secondary lymphoid organs (152, 153). Residual antigens may activate T- and B-lymphocytes, thereby resulting in persistent inflammation. The levels of pro-inflammatory markers in patients with long COVID remain elevated. Markers that are elevated during the acute phase including CRP and IL, may remain high 6 months or longer after disease onset (126, 154, 155). In the early recovery phase of COVID-19, patients who continued to develop PACS generally had higher levels of inflammatory biomarkers including TNF-α and interferon-inducible protein 10 (IP-10). PACS patients had an inflammatory state characterized by the up-regulation of TNF-α, IL-6, and IL-13, with decreased levels of IP-10 (156, 157). The levels of IL-17 and IL-2 were higher in the long COVID patient group, while higher levels of IL-10, IL-6, and IL-4 were shown in patients without sequelae (158, 159). Clinical manifestations of PACS correlate with the persistent pro-inflammatory phenotype induced by SARS-CoV-2 infection. We have elaborated on how inflammation triggers the activation of the coagulation cascade, eventually leading to thrombin-mediated fibrin generation and obstructive clot formation in the acute phase of COVID-19. Therefore, systemic inflammation may also be involved in Long COVID thrombosis. The presence of long-term systemic inflammation indicates that anti-inflammatory drugs could be useful not only during the acute phase of infection, but also in long COVID.

## Treatment strategy

### Antiviral therapy

A hyper-inflammatory response triggered by SARS-CoV-2 leads to immunothrombosis, thus early effective antiviral therapies are necessary for COVID-19 patients to prevent thrombus formation. Since its discovery, Omicron variant has rapidly increased in prevalence. Compared with Delta, Omicron has a significantly reduced risk of severe outcomes, with a higher reduction in the risk of serious consequences at the more severe endpoints (160). The antiviral monoclonal antibodies currently under investigation include molnupiravir, sotrovimab, and casirivimab-imdevimab. Molnupiravir is effective against Alpha and Beta variants in hamster and human cell models (*in vivo*), and against Delta and Omicron in studies *in vitro* (no *in vivo* data) (161). Sotrovimab retains its activity on Omicron, but requires higher concentrations to neutralize the virus. Casirivimab-imdevimab lacks efficacy against Omicron ba1variant (162, 163). Consequently, casirivimab-imdevimab is no longer recommended for COVID-19 treatment, unless infection with an earlier SARS-CoV-2 variant is confirmed (164, 165).

### Anticoagulant therapy

A large-scale observational study has shown that prophylactic doses of enoxaparin can improve the intubation rate and survival of hospitalized patients with COVID-19 (1). In large randomized controlled trials of hospitalized patients with COVID-19, the incidence of VTE in patients given prophylactic doses of anticoagulant therapy ranges from 6% to10%, and an incidence of 4-8% with therapeutic doses of anticoagulant therapy (166–168). In a prospective study of 803 hospitalized patients with COVID-19, increased doses of anticoagulant agents determined by the combination of disease severity, body weight, and D-dimer levels is associated with reduced mortality (169). In another observational study of 4,389 COVID-19 patients, analyses suggest more pronounced benefit with therapeutic compared to prophylactic dosing in anticoagulant therapy (170). In non-critically ill COVID-19 patients, therapeutic-dose anticoagulant therapy with heparin increases the probability of survival to hospital discharge and reduces the use of cardiovascular or respiratory organ support compared with thromboprophylaxis (1). These evidences indicate that therapeutic-dose anticoagulant therapy is superior to prophylactic-dose anticoagulant therapy in increasing the probability of hospital discharge and reducing mortality. However, recent studies do not favor the use of high dose of anticoagulant therapy (therapeutic-dose or full-dose anticoagulant therapy) in critically ill patients with COVID-19 (171, 172). The efficacy of anticoagulant treatment depends on the time of therapy initiation. As the disease progresses, anticoagulant therapeutic effect may be not satisfactory owing to the interactions of inflammation with the coagulation cascade. Full-dose anticoagulant therapy cannot reverse established disease process (thrombus formation) at the advanced stage (173, 174). Additionally, thrombosis leads to the consumption of coagulation factors, and full-dose anticoagulant therapy conversely worsens bleeding risk. Therefore, early administration of therapeutic-dose anticoagulant therapy can effectively reduce the incidence of thrombosis. Prophylactic anticoagulant therapy for COVID-19 patients after discharge remains controversial. However, post-discharge prophylactic anticoagulant treatment is beneficial to patients with a high risk of VTE. In patients at high risk of VTE discharged after hospitalisation due to COVID-19, thromboprophylaxis with rivaroxaban 10 mg/day for 35 days improved clinical outcomes compared with unextended thromboprophylaxis (175).

Thrombotic events are considered to be major causes of COVID-19 mortality, and current guidelines recommend the use of heparin for thromboprophylaxis and anticoagulant therapy (176). Heparin, including unfractionated heparin and low-molecular-weight heparin (LMWH), can bind to antithrombin (AT), and enhance inhibitory effects of AT on FXa and thrombin (177, 178). Accumulating evidence suggests that heparin can regulate the inflammatory response in multiple ways, including inhibition of both selectin-induced neutrophil adhesion and the release of inflammatory mediators (179, 180). Recent studies address the relationship between heparin and SARS-CoV-2, considering whether heparin directly binds to the S protein of SARS-CoV-2, and exerts direct antiviral effects (181).

In COVID-19, FXII is an attractive and rational therapeutic target. FXII can induce BK activation, which leads to the activation of downstream complement and the production of inflammatory cytokines (182). FXII inhibitors block the NETs-mediated contact activation (intrinsic) pathway of coagulation, and have anti-inflammatory effects (183). The most advanced FXII inhibitors are fully human or humanized monoclonal antibodies (including 3F7). Another potential therapeutic target is FXI, and FXI inhibitors include IONIS416858, bay1213790, MAA868, etc (184, 185). High doses of IONIS416858 (an ASO targeting FXI) used to treat venous thrombosis outperformed enoxaparin without increased bleeding risk. Direct oral anticoagulants (DOACs) directly inhibit thrombin or FXa to achieve an anticoagulant effect. FXa inhibitor rivaroxaban has been shown to prevent ischemic events in patients with cardiovascular diseases, and also slow down the progression of established atherosclerosis (186, 187). In patients with atrial fibrillation, oral rivaroxaban anticoagulation reduced plasma IL-6 and CRP levels (188). Compared to standard prophylactic anticoagulant therapy, rivaroxaban for 30 days improved clinical outcomes in hospitalized patients with COVID-19 and elevated d-dimer levels (189). Promising results have also been found using FXa inhibitors (primarily apixaban) in preventing thrombotic complications and reducing bleeding risks in COVID-19 patients compared with LMWH (190). Relatively few studies of the thrombin inhibitor dabigatran for anticoagulant therapy in COVID-19 have been reported. Compared with warfarin, DOACs have a broader therapeutic index, and do not require regular monitoring. DOACs are associated with a lower risk of systemic thromboembolic events and intracranial hemorrhage. Additionally, in the case of excessive anticoagulation, DOACs-induced bleeding can be reversed (191, 192). However, DOACs are not a universally accepted approach because of their high price. Considering the role PS plays in COVID-19 thrombosis, it may be an effective target for emerging therapeutics. The milk-fat globule protein lactadherin has been shown to compete with FV and FVIII for PS binding sites and reduce procoagulant activity *in vitro* and clotting in mouse bleeding models, and may therefore be a potential therapy to suppress thrombus formation.

t-PA is used for thrombolysis (generally after the failure of heparin treatment), rescue of severely ill patients, or for the prevention of possible thrombosis (193, 194). Considering there have been few adverse events of fibrinolytic therapy, early and local application of tPA, combined with accurate anti-inflammatory interventions targeting cytokines and chemokines, can improve survival in severe COVID-19 patients with ARDS (194). For COVID-19 patients without ARDS or other severe complications, fibrinolytic therapy can significantly improve the prognosis (194, 195). Currently, case reports and case series show that t-PA has significant efficacy for severe COVID-19 ARDS patients, with improved PaO2/FiO2 and no bleeding complications (196, 197). In a recent group of cases, five COVID-19 patients with severe hypoxemia (PaO2 <80 mmHg) and d-dimer greater than 1.5 μg/mL, receiving thrombolytic therapy (25 mg t-PA intravenous injection for 2 hours, 25 mg continuous infusion for the next 22 hours) and subsequent continuous infusion of heparin, showed improvement in oxygen demand, and three patients avoided mechanical ventilation after t-PA injection (196). In another study, three severe COVID-19 patients with respiratory failure undergoing the treatment of t-PA had a transient improvement in respiratory status, and one patient achieved a durable response (197).

### Antiplatelet therapy

Activated platelets are one of the important sources of MPs, and their essential role in COVID-19 coagulation have gradually been confirmed. Three classes of drugs can be used as antiplatelet therapy (198). The first category is aspirin, which irreversibly inactivates platelet cyclooxygenase-1 (COX-1), thus effectively preventing the production of the platelet agonist thromboxane A2 (TXA2). The second class of drugs that inhibit platelet activation is P2Y12 receptor antagonists (including clopidogrel, etc). P2Y12 is a major ADP receptor on platelets, and ADP binds to the P2Y12 receptor on platelets, promoting platelet aggregation and activation (199). The last category of drugs is the FDA-approved PAR1 antagonist (vorapaxar). Besides its antithrombotic activity due to inhibition of platelet aggregation, dipyridamole has also been shown to have antiviral, anti-inflammatory, and antioxidant properties. A clinical trial is currently underway to assess the effects of dipyridamole in COVID-19 patients (200, 201).

Observational studies showed that the mortality of hospitalized COVID-19 patients who received antiplatelet therapy before hospitalization was lower (202, 203). In a multivariate analysis of a propensity-matched population of COVID-19 patients including age, sex, hypertension, diabetes, renal failure, and invasive ventilation, administration of aspirin was associated with a lower risk of in-hospital mortality (204). Antiplatelet therapy during COVID-19 in-hospital stays reduced the risk of mortality, and shortened the duration of mechanical ventilation without simultaneously increasing the risk of bleeding (205, 206). Antiplatelet therapy also improved the ventilation/perfusion ratio in COVID-19 patients with severe respiratory failure, the mechanisms of which were the regulation of megakaryocyte function and platelet adhesion, and the prevention and intervention of pulmonary capillary thrombosis (207).

### Targeting nets

Targeting components of NETs are considered as potential interventions for COVID-19, the best known of which is Cl-amidine, an inhibitor of protein-arginine deiminase 4 and the NE inhibitor sivelestat (ONO-5046) (208). Cl-amidine was shown to inhibit NET release in both SARS-CoV-2- infected healthy neutrophils and blood neutrophils of COVID-19 patients (66). Sivelestat can improve lung function and oxygen saturation in patients with ARDS (209). A new generation of NE inhibitors (Lonodelestat, Alvelestat, CHF6333 and Elafin) is currently in clinical trials. Exogenous recombinant deoxyribonuclease 1 has been shown to reduce the concentrations of cell-free DNA and NETs in plasma *in vitro* and is a promising treatment option. The treatment of recombinant human (rh)DNase 1 was associated with a reduction in pulmonary DNA-MPO complexes as well as improved oxygenation.

Beyond NETs as a treatment target, platelets can promote NET formation, and platelet-derived HMGB1 is considered as a key molecule in arterial and venous thrombosis (210–212). Inhibiting HMGB1 with BoxA reduced thrombus burden deep venous thrombosis and improved the outcome of ischemic stroke (210, 212). Additionally, EVs from virus-activated platelets are potent endogenous danger signals, and the blockade of C-type lectins is a promising strategy to attenuate virus-induced NETs formation and coagulopathy. Targeting platelet-neutrophil interactions with antibodies against P-selectin or PSGL-1 may be an interesting approach regarding platelet-mediated NET formation (213). Crizanlizumab is a monoclonal antibody against p-selectin that is currently being investigated for the treatment of COVID-19-related vascular disease (214). More research into the mechanisms of NET formation in COVID-19 may pave the way for novel therapeutic approaches.

### Anti-inflammatory treatment

Anticoagulant therapy alone cannot resolve inflammation-driven thrombosis. Therefore, combination therapies are often required for COVID-19 patients. Additionally, targeting inflammation to prevent thrombosis does not affect the hemostatic balance, and avoids bleeding risk with existing treatments. Anakinra is a recombinant human IL-1 receptor antagonist. In a study of approximately 600 COVID-19 pneumonia patients at risk of respiratory failure, anakinra significantly improved the survival rate and shortened the length of hospital stay (215). Recent meta- analyses have suggested that COVID-19 patients the C-reactive protein serum concentrations greater than 100 mg/L benefited most from anakinra treatment (216, 217). In other trials, the level of soluble urokinase plasminogen activator receptor (suPAR) was used to identify the risk of severe COVID-19 at early stages, and anakinra was found to substantially reduce progression to severe respiratory failure and 30-day mortality (218, 219). Tocilizumab, a humanized monoclonal antibody targeting IL-6R, is an immunosuppressive drug commonly used in the treatment of chronic rheumatoid arthritis (220, 221). In hospitalized COVID-19 patients, treatment with IL-6 receptor antagonist (IL-6RA) reduced all-cause mortality. The most evident beneficiaries from IL-6RA were COVID-19 patients on respiratory support, including both non-invasive ventilation and invasive mechanical ventilation (221). Anti-TNF-α therapy is associated with lower hospitalization rates and a reduction in severe COVID-19 (admitted to critical care units or death) (222). However, these inflammatory mediators fulfill a crucial role in host defense. Therefore, the risk must be weighed between the potential benefits of suppressing cytokine storm and reducing defenses against pathogens.

### Anti-complement therapy

Elevated levels of several complement factors (C3 and C5) are observed in the plasma of COVID-19 patients and these factors are correlated to disease severity (223, 224). In a non-randomized clinical trial, eculizumab (a C5 inhibitor) significantly improved 15-day survival and oxygenation in severe COVID-19 patients (225). C3 inhibitor AMY-101 has been used for COVID-19 patients and is currently being evaluated in several clinical trials. Narsoplimab (an antibody against MASP-2), inhibits the lectin pathway, and has shown efficacy in a small study of COVID-19 patients (226) (**Table 1**).

**Table 1:** Prevention and treatment strategies for COVID-19 patients.

Treatment	Drugs	Significance	Reference
Antiviral therapy	Molnupiravir	Molnupiravir is effective against delta and omicron *in vitro* studies (no *in vivo* data).	(161, 227)
	Sotrovimab	Sotrovimab retains its activity on omicron, but requires higher concentrations to neutralize.	(163, 228)
	Casirivimab-imdevimab	Casirivimab-imdevimab lacks efficacy against omicron ba1variant.	(164, 227)
Anticoagulant therapy	Heparin	Heparin can bind to anti-thrombin, and enhance inhibitory effects of AT on coagulation factor Xa and thrombin.	(177, 229)
FXII inhibitor	3F7	FXII inhibitor is considered as an attractive therapeutic target for severe COVID-19 patients.	(198)
FXI inhibitor	IONIS416858	Higher doses of IONIS-416858 are superior to enoxaparin in treatment of venous thromboembolism without increased risk of bleeding.	(230)
FXa inhibitor	Rivaroxaban	Rivaroxaban for 30 days improves clinical outcomes in hospitalized patients with COVID-19 and elevated d-dimer levels.	(186, 189)
	Apixaban	A beneficial signal is observed in preventing thrombotic complications and reducing bleeding risks in patients with FXa inhibitors (primarily apixaban) compared to LMWH.	(185, 231)
FIIa inhibitor	Dabigatran	Relatively few studies of thrombin inhibitor (dabigatran) for anticoagulation in COVID-19 have been reported.	(232)
Targeting PS	Lactadherin	Lactadherin inhibits coagulation by effectively competing the binding sites of FV and FVIII with PS.	(78)
Antiplatelet therapy	Aspirin	Antiplatelet drugs (mainly aspirin) improve clinical outcomes of COVID-19 patients.	(233)
	Clopidogrel	Antiplatelet therapy has protective effects and reduce the risk of in-hospital mortality in COVID-19.	(234)
	Vorapaxar	The growing recognition of endotheliitis and thrombosis in COVID-19 patients provides a strong incentive to determine the potential utility of PAR1 inhibitors to improve the outcome of such patients.	(235)
	Dipyridamole	Dipyridamole can exert antithrombotic, antiviral, and anti-inflammatory effects.	(200)
PAD4 inhibitor	Cl-amidine	Cl-amidine can inhibit NET release in both SARS-CoV-2- infected healthy neutrophils and blood neutrophils of COVID-19 patients.	(208)
NE inhibitor	Sivelestat	Sivelestat can improve lung function and oxygen saturation in patients with ARDS.	(209)
Targeting DNA	(Rh)DNase 1	The treatment of recombinant human (rh)DNase 1 was associated with a reduction in pulmonary DNA-MPO complexes and improved oxygenation.	(209)
Inhibiting HMGB1	BoxA	BoxA can reduce thrombus burden deep venous thrombosis and improved the outcome of ischemic stroke.	(210, 212)
Targeting p-selectin	Crizanlizumab	Crizanlizumab is a monoclonal antibody against p-selectin that is currently being investigated for the treatment of COVID-19-related vascular disease.	(214)
IL-1RA	Anakinra	Anakinra significantly reduces the progression to respiratory failure and 30-day mortality.	(216)
IL-6RA	Tocilizumab	COVID-19 hospitalized patients receiving IL-6RA reduce all-cause mortality.	(218)
TNF-α antagonists	Adalimumab	Anti-TNF-α therapy is associated with lower hospitalization and severe COVID-19 disease.	(220)
C5 inhibitor	Eculizumab	Compared with the standard-of-care pathway only, eculizumab can significantly improve 15-day survival and oxygenation.	(226)
C3 inhibitor	AMY -101	C3 inhibitor AMY-101 has been used for COVID-19 patients.	(54)
MASP inhibitor	Narsoplimab	Narsoplimab shows efficacy in a small study of COVID-19 patients.	(236)

COVID-19: Coronavirus Disease 2019; AT: Aantithrombin; FXII: Factor XII; FXI: Factor XI; PS: Phosphatidylserine; PAR1: Protease activated receptor 1; NET: Neutrophil extracellular trap; PAD4: protein-arginine deiminase 4; NE: Neutrophil elastase; MPO: Myeloperoxidase; HMGB1: high-mobility group box 1; IL-1RA: IL-1 receptor antagonist; IL-6RA: IL-6 receptor antagonist; TNF-α: Tumor necrosis factor-α; MASP: mannose-binding lectin-associated ser-ine protease.

## Conclusion

The COVID-19 pandemic has brought unprecedented challenges. This review summarizes the mechanisms of how inflammation triggers the coagulation cascade, with a more nuanced understanding of interactions between coagulation and inflammatory system, inflammation can be targeted for the prevention and treatment of VTE and to blunt hyper-inflammatory and pro-thrombotic states. Accumulating evidence suggests that anti-inflammatory therapy plays a beneficial role in COVID-19. However, an important consideration in anti-inflammatory therapy is to balance the benefits of mitigating cytokine storm and the risk of immunosuppression hampering viral clearance. In advanced critically ill patients, the positive feedback loop between inflammation and the coagulation cascade leads to thrombosis and depletion of coagulation factors, which anti-thrombotic therapy cannot reverse. Consequently, early anti-thrombotic therapy can reduce adverse outcomes and improve the prognosis of COVID-19 patients.

## Author contributions

HJ and JS wrote the draft. XW, MX, and LL reviewed the manuscript, and JS and VN revised the manuscript and JS provided funding support. All authors contributed to the article and approved the submitted version.

## Conflict of interest

The authors declare that the research was conducted in the absence of any commercial or financial relationships that could be construed as a potential conflict of interest.

## Publisher’s note

All claims expressed in this article are solely those of the authors and do not necessarily represent those of their affiliated organizations, or those of the publisher, the editors and the reviewers. Any product that may be evaluated in this article, or claim that may be made by its manufacturer, is not guaranteed or endorsed by the publisher.
